# Angiopoietin-Like Protein 4 May Be an Interplay Between Serum Uric Acid and Triglyceride-Rich Lipoprotein Cholesterol

**DOI:** 10.3389/fcvm.2022.863687

**Published:** 2022-05-31

**Authors:** Yani Peng, Die Hu, Qingting Luo, Daoquan Peng

**Affiliations:** ^1^Department of Metabolism and Endocrinology, The Second Xiangya Hospital, Central South University, Changsha, China; ^2^Department of Cardiovascular Medicine, The Second Xiangya Hospital, Central South University, Changsha, China; ^3^Research Institute of Blood Lipid and Atherosclerosis, Central South University, Changsha, China

**Keywords:** serum uric acid, angiopoietin-like protein 4, triglyceride rich lipoproteins, ATH – atherosclerosis, triglycerides

## Abstract

**Background:**

Although the available evidence has indicated a link between elevated serum uric acid (SUA) level and dyslipidemia, the potential contribution of SUA on lipid profiles remains unclear. Experimental and clinical studies have revealed several mechanisms through which high serum angiopoietin-like protein 4 (ANGPTL4) level exerts deleterious effects on lipid metabolism, but the role of ANGPTL4 in SUA-associated dyslipidemia has not been well studied, so far.

**Methods:**

A total of 80 subjects were classified into high SUA group (*n* = 40) and low SUA group (*n* = 40) by the median value of SUA in the whole study population. Serum ANGPTL4 levels were determined by enzyme-linked immunosorbent assays.

**Results:**

In our study, we observed that not only serum triglyceride level [1.03 (0.78, 1.50) mmol/L vs. 1.59 (1.18, 2.37) mmol/L, *p* = 0.001] but also serum triglyceride-rich lipoprotein cholesterol (TRL-C) level [0.38 (0.32, 0.45) mmol/L vs. 0.46 (0.34, 0.54) mmol/L, *p* = 0.012] were significantly elevated in high SUA group. Additionally, serum ANGPTL4 in high SUA group was higher than in low SUA group [15.81 (11.88, 20.82) ng/ml vs. 22.13 (17.88, 32.09) ng/ml, *p* = 0.000]. Moreover, in all subjects, TRL-C levels were positively associated with SUA (*r* = 0.26, *p* = 0.023, *n* = 80) and ANGPTL4 levels (*r* = 0.24, *p* = 0.036, *n* = 80). Using stepwise multiple regression analysis to adjust for potential confounders, SUA was discovered to be an independent contributor to serum ANGPTL4 (*p* = 0.023). At the same time, serum ANGPTL4 was an independent contributor to the level of TRL-C (*p* = 0.000). However, the correlation between SUA and TRL-C disappeared after controlling for ANGPTL4 level.

**Conclusion:**

Serum uric acid was positively correlated to TRL-C. ANGPTL4 may be an interplay between SUA and associated elevation of TRL-C.

## Introduction

Serum uric acid (SUA), formed from xanthine, is the inert product of human purine metabolism, and its level is maintained by the balance between the production and excretion of uric acid. Excessive uric acid production by the liver, adipose tissue or muscle, and markedly decreased excretion by the kidney bring about the hyperuricemia. Overwhelming epidemiologic studies have reported that SUA is closely related to various cardiovascular conditions, such as dyslipidemia, metabolic syndrome, hypertension, and coronary artery disease (CAD) (1–9). However, until this day the exact role of SUA in these diseases remains controversial. It is difficult to determine whether SUA is a cause or a consequence. Previous studies suggested that hyperuricemia is a result of metabolic dysregulation, such as diabetes (10) and hypertension (6, 9), but some other studies indicated that SUA *per se* could play a causal role in the pathogenesis of metabolic disruptions (11–15). Liu et al. have found that high SUA could directly induce lipid metabolism disturbance by upregulating lysophosphatidyl choline acyltransferase 3 in the liver, which could induce p-STAT3 inhibition and SREBP-1c activation (3). Correspondingly, early prevention of hyperuricemia may be helpful to alleviate dyslipidemia (16). Thiazide treatment, which could increase SUA levels would lead to hypertriglyceridemia, while the febuxostat and allopurinol treatment lowering SUA could improve lipid profile (17, 18), suggesting the potential effect of SUA on the regulation of lipid metabolism (19). Whether there is an unidentified factor serving as a bridge mediating the deleterious effects of SUA on dyslipidemia remains unclear.

Recently, genome-wide association studies have revealed that an *ANGPTL4* variant is associated with TG, triglyceride-rich lipoprotein cholesterol (TRL-C), and LDL-C levels and thus has considerable effects on cardiovascular risk (20, 21). Functional studies *in vitro* have identified that angiopoietin-like protein 4 (ANGPTL4) potently inhibits lipoprotein lipase (LPL) and hepatic lipase action (22–24). Using murine and non-human primate models, Dewey et al. (25) have further clarified that ANGPTL4 plays a role in plasma triglyceride and very low-density lipoprotein (VLDL) metabolism by inhibiting the LPL activity. Both triglyceride and the non-HDL-C level could be effectively reduced through anti-ANGPTL4 therapy, which is consistent with the results in *ANGPTL4*-inactivating mutations carriers. Interestingly, accumulating studies have found that circulating ANGPTL4 is greatly increased in patients with metabolic syndrome or diabetes and is highly associated with metabolic traits, which is similar to the association of SUA with risk of metabolic syndrome (26). Although these experimental and clinical studies have revealed that elevated circulating ANGPTL4 exerts deleterious effects on TRLs (VLDL, IDL, and chylomicrons) metabolism, it is necessary to further explore the interaction between circulating ANGPTL4, metabolic syndrome, and multiple metabolic traits, such as SUA, cholesterol, and glucose.

Previous studies have demonstrated that ANGPTL4 is highly expressed in liver and adipose tissue in humans, especially the subcutaneous adipose tissue (27). Moreover, it could be regulated by various nutrition statuses in a tissue-specific manner according to the different functions of tissues and their physiological requirements for lipids (26, 28). For example, fasting could increase the circulating ANGPTL4 while the fed state could lower the serum ANGPTL4 level by regulating the lipid-sensing peroxisome proliferator-activated receptors (PPARs) to stimulate the ANGPTL4 expression in adipose tissue, liver, or intestine (28). However, the regulation of ANGPTL4 in metabolic syndrome has not yet been extensively studied. Of note, SUA could also be produced and secreted from adipose tissue that had higher expression and activities of xanthine catabolic enzyme, xanthine oxidoreductase (XOR) (18). Additionally, several studies have also found that SUA production is related to lipolysis, adipogenesis, adipose tissue remodeling, and active lipid metabolism, indicating the potential link between SUA and the regulation of ANGPTL4 (29, 30).

The current study was designed to explore the relationship between SUA and ANGPTL4 and their independent effect on triglyceride-rich lipoprotein cholesterol.

## Materials and Methods

### Subjects

Our cohort consisted of 80 subjects who were enrolled in the department of Cardiology of The Second Xiangya Hospital, Central South University, Changsha, China. To observe the serum ANGPTL4 level and its relationship with lipid profiles and SUA levels, all subjects were categorized into high SUA group (*n* = 40) and low SUA group (*n* = 40) by the median value of SUA in the whole study population. Besides, we also classified all participants into CAD group (*n* = 45) and non-CAD group (*n* = 35) according to the clinical symptoms, ischemic changes in ECG, and CAG findings to investigate the serum ANGPTL4 levels and lipid profiles in patients with CAD. The detailed criteria for diagnosis of CAD and the exclusion criteria for participants were described before (31). All subjects gave informed consent, and the protocol was approved by an institutional review committee.

### Clinical and Biochemical Measurements

Patient information, such as age, gender, smoking history, and medication history, were recorded. Anthropometric measurements [height, weight, body mass index (BMI), and blood pressure] were assessed after overnight fasting for at least 10 h. Blood samples were collected after an overnight fast, and the samples were stored at −80°C until analysis. Lipid profile, such as total cholesterol (TC), triglyceride (TG), low-density lipoprotein cholesterol (LDL-C), high-density lipoprotein cholesterol (HDL-C), hepatic function, renal function, SUA, and high sensitive C reactive protein (hs-CRP), were evaluated by standard laboratory procedures. TRL-C has have simply been calculated as total cholesterol minus HDL-C and LDL-C levels. The estimated glomerular filtration rate (eGFR) was assessed by the Mayo formula (32).

### Measurement of Serum ANGPTL4 Levels

Serum ANGPTL4 concentration was determined by a sandwich enzyme-linked immunosorbent assay (ELISA). Commercial ELISA kits from CUSABIO Corporation (CSB-EL001712HU) were used to assess the concentrations of ANGPTL4.

### Statistical Analysis

Clinical data are presented as mean ± SD or median with the interquartile range as appropriate. Comparisons between categorical data were performed with *χ^2^* tests, while continuous variables were assessed by independent *t*-tests or one-way analysis of variance (for normal distribution) or non-parametric test (for skewed distribution). As skewed distributions, serum ANGPTL4 and SUA values from subjects were compared using the Mann-Whitney *U*-test. The original ANGPTL4 values were transformed logarithmically when it was described in Figures 2, 3B. The correlations between ANGPTL4 and SUA as well as blood lipids were tested by Spearman’s correlation coefficients. Stepwise multiple linear regression analysis was performed to determine the variables with the independent significant association among ANGPTL4 and SUA and blood lipid profile. These variables included all potential ones which might have a significant relationship with blood lipid profile and uric acid in univariate analyses. A two-tailed *p*-value < 0.05 was considered statistically significant. Statistical analysis was performed with Statistical Package for Social Sciences version 22.0 and plots were created with GraphPad Prism V.6.0 (GraphPad Software, Inc., La Jolla, CA, United States).

**FIGURE 1:**
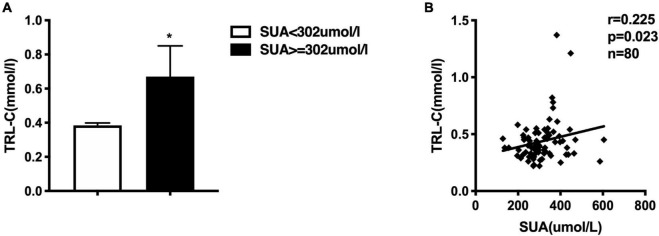
**(A)** Comparison of serum triglycerides-rich lipoprotein cholesterol (TRL-C) between high SUA group and low SUA group, the Mann-Whitney *U*-test showed that TRL-C levels were significantly greater in the high SUA group than in the low SUA group, *p < 0.05. **(B)** Serum uric acid is positively correlated to TRL-C concentration in all subjects.

**FIGURE 2:**
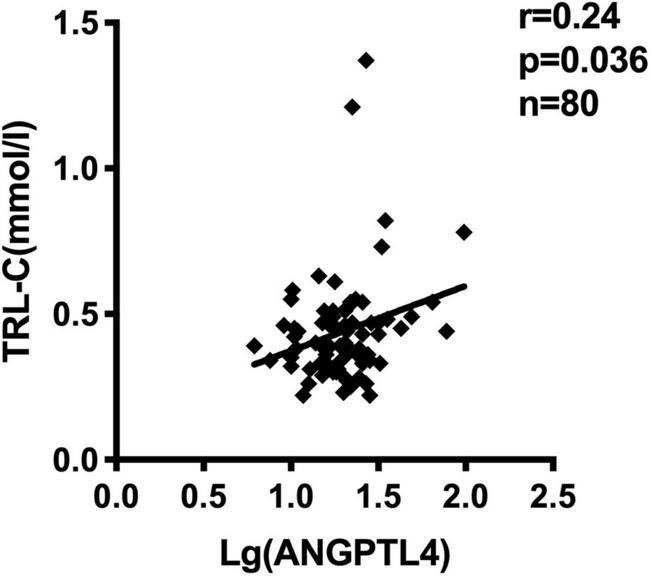
Serum ANGPTL4 (Log-transformed) concentration correlated to TRL-C concentration in all subjects (*n* = 80).

**FIGURE 3:**
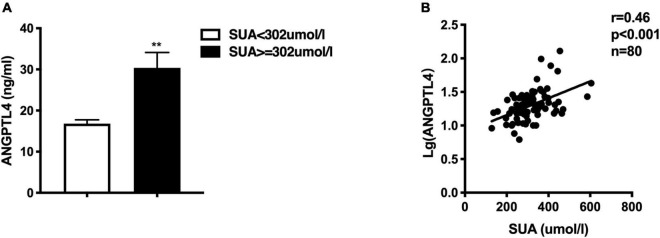
**(A)** Comparison of circulating ANGPTL4 between high SUA group and low SUA group, the Mann-Whitney *U*-test showed that serum ANGPTL4 was significantly greater in the high SUA group than in the low SUA group, **p < 0.01. **(B)** Serum uric acid is positively correlated to ANGPTL4 concentration in all subjects.

## Results

### The Relationship Between Lipid Profile and Serum Uric Acid Levels

Epidemiologic studies have depicted a well-established relationship between SUA concentration and CAD, dyslipidemia, and metabolic syndrome. In our study, we used the medium value of SUA in the whole study population (302.4 μmol/L) as the cut-off point to define the high or low SUA group, which is similar to previous epidemiological studies (19, 33). Table 1 presented the clinical and biochemical characteristics of subjects by this SUA cut-off value. As shown in Table 1, there were more men than women in the high SUA group. Consistent with previous reports (1, 6), individuals with higher SUA levels were more hypertensive and exhibited markedly higher values of serum hs-CRP. There is no significantly different distribution of patients with CAD between subjects with high and low-SUA levels. Consistent with previous reports (1, 2, 4, 5), individuals with high SUA levels (≥302.4 μmol/l) have significantly higher TG levels [1.03 (0.78, 1.50) mmol/L vs. 1.59 (1.18, 2.37) mmol/L, *p* = 0.001] and TRL-C levels [0.38 (0.32, 0.45) vs. 0.46 (0.34, 0.54) mmol/L, *p* = 0.012; Figure 1A]. Meanwhile, the HDL-C level in the high-SUA group was lower than that in the low-SUA group [1.20 (1.10, 1.30) mmol/L vs. 1.00 (0.80, 1.10) mmol/L, *p* = 0.000]. The comparisons were also made between CAD and non-CAD groups in terms of metabolic parameters. Our data showed that patients with CAD tend to have higher TRL-C [0.38 (0.30, 0.47) mmol/L vs. 0.44 (0.35, 0.51) mmol/L, *p* = 0.045] and lower HDL-C [1.20 (1.00, 1.33) mmol/L vs. 1.01 (0.86, 1.19) mmol/L, *p* = 0.021] compared to the control group. There was no significant difference in LDL-C between patients with CAD and control, possibly due to more statin users in the CAD group (58%) than in controls (9%). Unexpectedly, despite patients with CAD tend to have higher angiopoietin-like protein 4 (ANGPTL4) concentrations and SUA levels than non-CAD subjects, the difference between the two groups did not achieve statistical significance (Table 2).

**TABLE 1:** Clinical characteristics of the study population according to the median value of SUA levels (302 μmol/l).

SUA	< 302 μmol/l	≥302 μmol/l	*P* value
	(*n* = 40)	(*n* = 40)	
Age, years	59.28 ± 6.99	58.13 ± 7.33	0.475
Gender, male/female	17/23	29/11	**0.007**
BMI (Kg/m^2^)	23.38 ± 3.08	24.57 ± 3.72	0.126
Current smokers, n (%)	17 (43)	24 (60)	0.117
Statin users, n (%)	13 (33)	16 (40)	0.485
Hypertension, n (%)	12 (30)	20 (50)	0.068
CAD, n (%)	19 (48)	26 (65)	0.115
eGFR (mL/min/1.73 m^2^)	102.23 ± 13.22	104.69 ± 12.65	0.397
hsCRP	0.80 (0.44, 4.71)	2.04 (1.21, 8.60)	**0.031**
TC (mmol/l)	4.10 (3.41, 4.32)	4.09 (3.46, 4.83)	0.672
TG (mmol/l)	1.03 (0.78, 1.50)	1.59 (1.18, 2.37)	**0.001**
HDL-C (mmol/l)	1.20 (1.10, 1.30)	1.00 (0.80, 1.10)	**0.000**
LDL-C (mmol/l)	2.43 (1.89, 2.83)	2.62 (1.90, 3.09)	0.299
TRL-C (mmol/l)	0.38 (0.32, 0.45)	0.46 (0.34, 0.54)	**0.012**
ANGPTL4 (ng/ml)	15.81 (11.88, 20.82)	22.13 (17.88, 32.09)	**0.000**

*BMI, body mass index; eGFR, estimated glomerular filtration rate; hs-CRP, high sensitive C reactive protein; TG, triglycerides; TC, total cholesterol; HDL-C, high-density lipoprotein cholesterol; LDL-C, low-density lipoprotein cholesterol; TRL-C, triglyceride-rich lipoprotein-cholesterol; SUA, serum uric acid; ANGPTL4, angiopoietin-like 4. The bold values mean these parameters had statistical significance.*

**TABLE 2:** Clinical characteristics of the CAD group and non-CAD group.

	non-CAD group (*n* = 35)	CAD group (*n* = 45)	*P* value
Age, years	58.14 ± 7.87	59.13 ± 6.57	0.541
Gender, male/female	20/15	26/19	0.955
BMI (Kg/m^2^)	23.40 ± 4.10	24.40 ± 2.83	0.201
Current smokers, n (%)	14 (40)	27 (60)	0.076
Statin users, n (%)	3 (9)	26 (58)	0.000
Hypertension, n (%)	11 (31)	21 (47)	0.168
eGFR (mL/min/1.73 m^2^)	105.13 ± 13.64	102.16 ± 12.32	0.309
hsCRP	1.60 (0.50, 4.75)	1.83 (0.92, 8.76)	0.272
TG (mmol/l)	1.22 (0.77, 1.72)	1.44 (0.95, 2.15)	0.063
TC (mmol/l)	4.14 (3.62, 4.59)	4.07 (3.29, 4.66)	0.535
HDL-C (mmol/l)	1.20 (1.00, 1.33)	1.01 (0.86, 1.19)	0.021
LDL-C (mmol/l)	2.60 (2.02, 3.01)	2.51 (1.84, 2.99)	0.541
TRL-C (mmol/l)	0.38 (0.30, 0.47)	0.44 (0.35, 0.51)	0.045
SUA (μmol/l)	293.50 (263.80, 339.40)	325.20 (257.60, 371.20)	0.271
ANGPTL4 (ng/ml)	19.04 (12.63, 25.69)	20.22 (15.32, 26.51)	0.412

*BMI, body mass index; eGFR, estimated glomerular filtration rate; hs-CRP, high sensitive C reactive protein; TG, triglycerides; TC, total cholesterol; HDL-C, high-density lipoprotein cholesterol; LDL-C, low-density lipoprotein cholesterol; TRL-C, triglyceride-rich lipoprotein-cholesterol; SUA, serum uric acid; ANGPTL4, angiopoietin-like 4.*

Furthermore, we also investigated the correlation between SUA and lipid profile. As described in Table 3, SUA level was positively correlated with TG (*r* = 0.286, *p* = 0.01), TRL-C (*r* = 0.225, *p* = 0.023, Figure 1B), BMI (*r* = 0.312, *p* = 0.005), as well as serum hs-CRP level (*r* = 0.289, *p* = 0.038) but negatively related to HDL-C level (*r* = −0.470, *p* = 0.000). Considering weight as a potential contributor to SUA-associated dyslipidemia, we adjusted for BMI and found that the relationship between SUA levels and TRL-C or HDL-C remained significant but the association between SUA and TG disappeared.

**TABLE 3:** Correlation analysis of SUA and cardio-metabolic parameters.

	SUA	
Variables	*r*	*p*
TC	0.074	0.515
**TG**	**0.286**	**0.010**
LDL-C	0.098	0.385
**HDL-C**	−**0.470**	**0.000**
**TRL-C**	**0.255**	**0.023**
**hs-CRP**	**0.289**	**0.038**
**BMI**	**0.312**	**0.005**

*TG, triglycerides; TC, total cholesterol; HDL-C, high-density lipoprotein cholesterol; LDL-C, low-density lipoprotein cholesterol; TRL-C, triglyceride-rich lipoprotein-cholesterol; SUA, serum uric acid; hs-CRP, high sensitive C reactive protein; BMI, body mass index. The bold values mean these parameters had statistical significance.*

### Association of Circulating Angiopoietin-Like Protein 4 With Lipid Profile

ANGPTL4, as a lipoprotein lipase inhibitor, plays a key role in triglycerides metabolism (28). Current studies have shown inconsistent data about the relationship between serum ANGPTL4 and lipid parameters (27). In our study, we observed that serum ANGPTL4 concentrations were positively associated with total cholesterol (*r* = 0.254, *p* = 0.023) and TRL-C (*r* = 0.235, *p* = 0.036, Figure 2) but not with LDL-C, triglycerides, and HDL-C (Table 4). Meanwhile, we analyzed the relationship of TRL-C with some metabolic factors. As presented in Table 5, the level of TRL-C was significantly correlated with both the concentration of SUA and ANGPTL4. However, in the multiple regression analysis, the contribution of SUA to TRL-C level disappeared but the contribution of hs-CRP to TRL-C appeared after controlling for the effect ofANGPTL4 and other related factors, such as age, gender, statin therapy, and estimated glomerular filtration rate (eGFR), leaving ANGPTL4 an independent contributor to TRL-C level (Table 6).

**TABLE 4:** Correlation analysis of ANGPTL4 and cardio-metabolic parameters.

	ANGPTL4	
Variables	*r*	*p*
**TC**	**0.254**	**0.023**
TG	0.168	0.136
LDL-C	0.170	0.131
HDL-C	−0.117	0.301
**TRL-C**	**0.235**	**0.036**
**hs-CRP**	**0.445**	**0.001**
BMI	0.061	0.596

*TG, triglycerides; TC, total cholesterol; HDL-C, high-density lipoprotein cholesterol; LDL-C, low-density lipoprotein cholesterol; TRL-C, triglyceride-rich lipoprotein-cholesterol; SUA, serum uric acid; hs-CRP, high sensitive C reactive protein; BMI, body mass index. The bold values mean these parameters had statistical significance.*

**TABLE 5:** Correlation analysis of TRL-C and metabolic factors.

	TRL-C	
Variables	*r*	*p*
BMI	0.114	0.317
eGFR	0.010	0.928
hs-CRP	0.164	0.259
**SUA**	**0.255**	**0.023**
**ANGPTL4**	**0.235**	**0.036**

*BMI, body mass index; eGFR, estimated glomerular filtration rate; hs-CRP, high sensitive C reactive protein; ANGPTL4, angiopoietin-like 4; SUA, serum uric acid; TRL-C, triglyceride-rich lipoprotein-cholesterol. The bold values mean these parameters had statistical significance.*

**TABLE 6:** Stepwise multiple regression analysis detecting independent contributor to TRL-C.

Factor	B	β	*P* value
age	−0.031	−0.196	0.122
sex	−0.454	−0.204	0.173
Statin	0.119	0.056	0.567
eGFR	0.022	0.223	0.134
BMI	0.013	0.056	0.535
Hypertension	−0.005	−0.012	0.908
**hs-CRP**	−**0.046**	−**0.012**	**0.021**
**ANGPTL4**	**0.044**	**0.900**	**0.000**
SUA	0.000	0.008	0.944

*Sex: female = 0, male = 1; Statin: using statin = 1, without using statin = 0; eGFR, estimated glomerular filtration rate; hs-CRP, high sensitive C reactive protein; BMI, body mass index; SUA, serum uric acid; TRL-C, triglyceride-rich lipoprotein cholesterol; ANGPTL4, angiopoietin-like 4; B, unstandardized coefficients; β, standardized regression coefficients. The bold values mean these parameters achieved statistical significance.*

Considering the role of inflammation status in dyslipidemia and serum ANGPTL4 level (34, 35), we also assessed the relationship between the serum ANGPTL4 and hs-CRP, an established marker of inflammation. In accordance with previous reports (34), the serum hs-CRP levels had a strong correlation with serum ANGPTL4 levels (*r* = 0.445, *p* = 0.001, Table 4).

### The Relationship Between Serum Uric Acid and ANGPTL4

Given the observance that individuals with high SUA levels have significantly higher serum TG and TRL-C levels, we further assessed the circulating ANGPTL4 in the high SUA group. Interestingly, compared to the low SUA group, higher ANGPTL4 was found in the high SUA group [15.81 (11.88, 20.82) ng/ml vs. 22.13 (17.88, 32.09) ng/ml, *p* = 0.000, Figure 3A]. In addition, serum ANGPTL4 concentrations were positively correlated with SUA levels in all subjects (*r* = 0.46, *p* = 0.000, Figure 3B). More importantly, multiple regression analysis revealed that the association between ANGPTL4 and SUA level was independent of age, gender, statin, BMI, hypertension, and eGFR as well as serum hs-CRP levels, indicating that SUA is a potential contributor to ANGPTL4 (Table 7). To investigate the relationship of serum ANGPTL4 in SUA-associated dyslipidemia, we analyzed the correlation between ANGPTL4 and TRL-C in the high SUA group and the low SUA group after adjusting the confounders (sex, gender, and BMI), respectively. We found a strong correlation between ANGPTL4 and TRL-C only in the high SUA group (*r* = 0.717, *p* = 0.000) and not in subjects with low SUA levels (*r* = −0.88, *p* = 0.604), indicating ANGPTL4 may play a role in TRL-C metabolism in patients with hyperuricemia and thus mediate SUA-associated dyslipidemia.

**TABLE 7:** Stepwise multiple regression analysis detecting independent contributor to ANGPTL4.

Factor	B	β	*P* value
Age	0.356	0.110	0.567
Sex	10.808	0.237	0.294
Statin	−4.697	−0.109	0.469
eGFR	−0.370	−0.181	0.419
BMI	0.578	0.075	0.653
Hypertension	−10.188	−0.238	0.146
hs-CRP	0.875	0.231	0.145
**SUA**	**0.107**	**0.375**	**0.023**

*Sex: female = 0, male = 1; Statin: using statin = 1, without using statin = 0; eGFR, estimated glomerular filtration rate; hs-CRP, high sensitive C reactive protein; BMI, body mass index; SUA, serum uric acid; TRL-C, triglyceride-rich lipoprotein cholesterol; ANGPTL4, angiopoietin-like 4; B, unstandardized coefficients; β, standardized regression coefficients. The bold values mean these parameters had statistical significance.*

## Discussion

Here, we discovered serum uric acid (SUA) to be highly associated with multiple features of the metabolic syndrome, such as triglycerides and BMI, and negatively associated with HDL-C levels, which is consistent with previous epidemiologic studies (1, 2, 4, 7, 8, 10). Apart from this, our study has also shown that in the high SUA group, not only triglyceride but also triglyceride-rich lipoproteins cholesterol (TRL-C) are markedly elevated. Moreover, TRL-C level is positively correlated with SUA level, suggesting a complex interaction between SUA and TRL-C metabolism. SUA had formerly been regarded as an “innocent bystander,” where its elevation was believed to be attributed to the influence of insulin resistance in metabolic syndrome which was accompanied by dyslipidemia. However, recent evidence supports the possibility that an elevated uric acid level may play a role in the pathogenesis of dyslipidemia, metabolic syndrome, hypertension, and atherosclerosis via insulin resistance dependent or independent pathway (3, 11, 12, 15).

Bearing in mind that a potential “bridge” could be mediating the interplay between SUA and lipid metabolism, we further looked into ANGPTL4 levels in our patient population. Our study first provided evidence that circulating ANGPTL4 levels were significantly elevated in individuals with high SUA levels. We also observed that circulating ANGPTL4 level was independently associated with SUA level even after adjustment for serum hs-CRP which has been reported to participate in dyslipidemia and increase ANGPTL4 expression in metabolic syndrome (34), suggesting a potential contribution from SUA in ANGPTL4 regulation. Previous studies have revealed that ANGPTL4 is highly expressed and regulated in adipose tissue by the response to variations in the nutritional state (36, 37). Intriguingly, Tsushima et al. (18) reported that SUA also could be produced and secreted from adipose tissue through xanthine catabolic enzyme–xanthine oxidoreductase (XOR). XOR could regulate the activity of PPARγ, which is reported as a transcription factor of ANGPTL4 in human adipose tissue (27, 29). Moreover, a study has revealed that ANGPTL4 is highly expressed in the omental and subcutaneous adipose tissue which was the major origin of SUA (27). Besides, some studies have indicated that in obesity SUA is related to lipolysis by some unknown mechanism (18). Taking this into consideration, the present results indicated further investigation is required to clarify the underlying mechanism whereby SUA modulates ANGPTL4 levels in humans.

Recent studies have reported that circulating ANGPTL4 is strongly related to lipid and multiple features of the metabolic syndrome (21, 26). In our study, we observed that serum ANGPTL4 is negatively correlated with HDL-C level but still failed to observe a significant positive correlation of ANGPTL4 with triglyceride, which is consistent with previous studies (38, 39). However, we incidentally found that serum ANGPTL4 levels were independently correlated with TRL-C levels, especially in subjects with hyperuricemia. *In vivo* and *in vitro* studies have confirmed that ANGPTL4 could inhibit the activity of lipoprotein lipase (LPL) and thus regulate triglyceride hydrolysis and plasma triglyceride-rich lipoproteins (TRLs) clearance (38), which may partially explain the role of ANGPTL4 on TRLs metabolism. Indeed, the function of ANGPLT4 is dependent on the tissue requirement for lipid, so different metabolic states would interfere with ANGPLT4-mediated lipid metabolism. Some studies have revealed that ANGPTL4 stimulates intracellular lipolysis in adipocytes and myocytes in the fasting state. The metabolism of TG-rich remnant particles is not completely dependent on LPL activity, the process of receptor-mediated endocytosis of remnant particles and intracellular lipolysis is also important (38). Meanwhile, some studies have shown that liver-derived ANGPTL4 could not only regulate the LPL activity but also inhibit hepatic lipase from hydrolyzing the lipid cargo of TG-rich remnant particles and thus have an influence on remnant cholesterol metabolism (40). Therefore, whether hyperuricemia could affect the function of ANGPLT4 is another interesting question that will be explored in our future study.

In addition, in our investigation, although circulating ANGPTL4 concentrations tended to be greater in the CAD group, we found it to fall short of statistical significance. According to published clinical studies, ANGPTL4 levels in patients with CAD vary widely despite the strong genetic evidence for the role of ANGPTL4 on CAD risk (21, 41, 42). We know from clinical and animal studies that ANGPTL4 can be influenced by many factors, such as the metabolic state (22), abnormal lipid profiles (43), and feedback loops, with other members of the angiopoietin-like protein family-like ANGPTL3 (44) and ANGPTL8 (26, 38), making it difficult to interpret its changes in the serum.

The limitations of our study are: first, a relatively small number of subjects may not represent the observed findings for the entire population. Additionally, our study is a cross-sectional study that does not allow us to ensure complete control of all the potential (still unknown)confounding factors. Indeed, we could not rule out the possibility that some key parameters, such as glucose, insulin, and glucagon, may attenuate the relationship between the ANGPTL4 and SUA-associated dyslipidemia. Moreover, the correlations between SUA and TRL-C may be confounded by other factors due to the complex regulation of TRL-C metabolism, such as post heparin plasma lipoprotein lipase (LPL) activity, ApoC3, and ANGPTL3/8, which were not evaluated in our study and known to affect lipid metabolism. However, the present study findings are still worthy of a reference. Large-scale prospective studies investigating the association between hyperuricemia and ANGPTL4 are required to replicate our findings. To validate the relationship between SUA and ANGPTL4, a follow-up study of patients with higher levels of SUA treated with uric acid-lowering agent would be most helpful. Meanwhile, related mechanistic studies to confirm the cause-and-effect relationships between SUA and ANGPTL4 remain to be explored in the future.

In conclusion, our current study exhibited that individuals with high SUA tend to have higher circulating ANGPTL4 levels as well as increased TRL-C. SUA was an independent contributor to ANGPTL4, which prompts our future work to determine whether SUA could directly impact ANGPTL4 expression and lowering SUA could subsequently decrease ANGPTL4 and improve ANGPTL4-mediated lipid disorders. Also, this interaction between ANGPTL4 and SUA-related lipid disturbance creates the possibility for ANGPTL4-targeted therapies to impart a significant benefit in hyperuricemia-associated dyslipidemia.

## Data Availability Statement

The raw data supporting the conclusions of this article will be made available by the authors, without undue reservation.

## Ethics Statement

The studies involving human participants were reviewed and approved by the study protocol conforms to the Ethical Guidelines of the 1975 Declaration of Helsinki as reflected in *a priori* approval by the Medical Ethics Committee of the Second Xiangya Hospital of Central South University. The patients/participants provided their written informed consent to participate in this study.

## Author Contributions

YP, DH, and DP designed the study and performed the data analysis. DH and QL performed the experiments. YP and DH prepared the manuscript. All authors read and approved the final manuscript.

## Conflict of Interest

The authors declare that the research was conducted in the absence of any commercial or financial relationships that could be construed as a potential conflict of interest.

## Publisher’s Note

All claims expressed in this article are solely those of the authors and do not necessarily represent those of their affiliated organizations, or those of the publisher, the editors and the reviewers. Any product that may be evaluated in this article, or claim that may be made by its manufacturer, is not guaranteed or endorsed by the publisher.

## Abbreviations

CAD: coronary artery disease
ANGPTL4: angiopoietin-like protein 4
HDL: high-density lipoprotein
LDL: low-density lipoprotein
TG: triglyceride
TC: total cholesterol
non-HDL-C: non-high-density lipoprotein cholesterol
BMI: body mass index
BUN: blood urea nitrogen
SUA: serum uric acid
CR: creatinine
LPL: lipoprotein lipase
hs-CRP: high sensitive C reactive protein
eGFR: estimated glomerular filtration rate.

## References

[1] Ali N Rahman S Islam S Haque T Molla NH Sumon AH The relationship between serum uric acid and lipid profile in Bangladeshi adults. *BMC Cardiovasc Disord.* (2019) 19:42. 10.1186/s12872-019-1026-2 30791868 PMC6385393

[2] Peng TC Wang CC Kao TW Chan JY Yang YH Chang YW Relationship between hyperuricemia and lipid profiles in US adults. *Biomed Res Int.* (2015) 2015:127596. 10.1155/2015/127596 25629033 PMC4299312

[3] Liu N Sun Q Xu H Yu X Chen W Wei H Hyperuricemia induces lipid disturbances mediated by LPCAT3 upregulation in the liver. *Faseb J.* (2020) 34:13474–93. 10.1096/fj.202000950R 32780898

[4] Son M Seo J Yang S. Association between dyslipidemia and serum uric acid levels in Korean adults: Korea National Health and Nutrition Examination Survey 2016-2017. *PLoS One.* (2020) 15:e0228684. 10.1371/journal.pone.0228684 32059030 PMC7021293

[5] Zhang S Wang Y Cheng J Huangfu N Zhao R Xu Z Hyperuricemia and cardiovascular disease. *Curr Pharm Des.* (2019) 25:700–9. 30961478 10.2174/1381612825666190408122557

[6] Sanchez-Lozada LG Rodriguez-Iturbe B Kelley EE Nakagawa T Madero M Feig DI Uric acid and hypertension: an update with recommendations. *Am J Hypertens.* (2020) 33:583–94. 10.1093/ajh/hpaa044 32179896 PMC7368167

[7] Cibičková L Langová K Vaverková H Kubíčková V Karásek D. Correlation of uric acid levels and parameters of metabolic syndrome. *Physiol Res.* (2017) 66:481–7. 10.33549/physiolres.933410 28248530

[8] Kocak MZ Aktas G Erkus E Sincer I Atak B Duman T. Serum uric acid to HDL-cholesterol ratio is a strong predictor of metabolic syndrome in type 2 diabetes mellitus. *Rev Assoc Med Bras (1992).* (2019) 65:9–15. 10.1590/1806-9282.65.1.9 30758414

[9] Kosekli MA Kurtkulagii O Kahveci G Duman TT Tel BMA Bilgin S The association between serum uric acid to high density lipoprotein-cholesterol ratio and non-alcoholic fatty liver disease: the abund study. *Rev Assoc Med Bras (1992).* (2021) 67:549–54. 10.1590/1806-9282.20201005 34495059

[10] Bonakdaran S Kharaqani B. Association of serum uric acid and metabolic syndrome in type 2 diabetes. *Curr Diabetes Rev.* (2014) 10:113–7. 10.2174/1573399810666140228160938 24588601

[11] King C Lanaspa MA Jensen T Tolan DR Sánchez-Lozada LG Johnson RJ. Uric acid as a cause of the metabolic syndrome. *Contrib Nephrol.* (2018) 192:88–102. 10.1159/000484283 29393133

[12] Lima WG Martins-Santos ME Chaves VE. Uric acid as a modulator of glucose and lipid metabolism. *Biochimie.* (2015) 116:17–23. 10.1016/j.biochi.2015.06.025 26133655

[13] Katsiki N Dimitriadis GD Mikhailidis DP. Serum Uric acid and diabetes: from pathophysiology to cardiovascular disease. *Curr Pharm Des.* (2021) 27:1941–51. 10.2174/1381612827666210104124320 33397230

[14] Chen S Chen D Yang H Wang X Wang J Xu C. Uric acid induced hepatocytes lipid accumulation through regulation of miR-149-5p/FGF21 axis. *BMC Gastroenterol.* (2020) 20:39. 10.1186/s12876-020-01189-z 32070295 PMC7027271

[15] Xie D Zhao H Lu J He F Liu W Yu W High uric acid induces liver fat accumulation via ROS/JNK/AP-1 signaling. *Am J Physiol Endocrinol Metab.* (2021) 320:E1032–43. 10.1152/ajpendo.00518.2020 33900847

[16] Castro VMF Melo AC Belo VS Chaves VE. Effect of allopurinol and uric acid normalization on serum lipids hyperuricemic subjects: a systematic review with meta-analysis. *Clin Biochem.* (2017) 50:1289–97. 10.1016/j.clinbiochem.2017.07.013 28754333

[17] George J Carr E Davies J Belch JJ Struthers A. High-dose allopurinol improves endothelial function by profoundly reducing vascular oxidative stress and not by lowering uric acid. *Circulation.* (2006) 114:2508–16. 10.1161/circulationaha.106.651117 17130343

[18] Tsushima Y Nishizawa H Tochino Y Nakatsuji H Sekimoto R Nagao H Uric acid secretion from adipose tissue and its increase in obesity. *J Biol Chem.* (2013) 288:27138–49. 10.1074/jbc.M113.485094 23913681 PMC3779712

[19] Feig DI Kang DH Johnson RJ. Uric acid and cardiovascular risk. *N Engl J Med.* (2008) 359:1811–21. 18946066 10.1056/NEJMra0800885 PMC2684330

[20] Helgadottir A Gretarsdottir S Thorleifsson G Hjartarson E Sigurdsson A Magnusdottir A Variants with large effects on blood lipids and the role of cholesterol and triglycerides in coronary disease. *Nat Genet.* (2016) 48:634–9. 10.1038/ng.3561 27135400 PMC9136713

[21] Muendlein A Saely CH Leiherer A Fraunberger P Kinz E Rein P Angiopoietin-like protein 4 significantly predicts future cardiovascular events in coronary patients. *Atherosclerosis.* (2014) 237:632–8. 10.1016/j.atherosclerosis.2014.10.028 25463098

[22] Mandard S Zandbergen F van Straten E Wahli W Kuipers F Muller M The fasting-induced adipose factor/angiopoietin-like protein 4 is physically associated with lipoproteins and governs plasma lipid levels and adiposity. *J Biol Chem.* (2006) 281:934–44. 10.1074/jbc.M506519200 16272564

[23] Koster A Chao YB Mosior M Ford A Gonzalez-DeWhitt PA Hale JE Transgenic angiopoietin-like (angptl)4 overexpression and targeted disruption of angptl4 and angptl3: regulation of triglyceride metabolism. *Endocrinology.* (2005) 146:4943–50. 10.1210/en.2005-0476 16081640

[24] Desai U Lee EC Chung K Gao C Gay J Key B Lipid-lowering effects of anti-angiopoietin-like 4 antibody recapitulate the lipid phenotype found in angiopoietin-like 4 knockout mice. *Proc Natl Acad Sci U S A.* (2007) 104:11766–71. 10.1073/pnas.0705041104 17609370 PMC1913890

[25] Dewey FE Gusarova V O’Dushlaine C Gottesman O Trejos J Hunt C Inactivating variants in ANGPTL4 and risk of coronary artery disease. *N Engl J Med.* (2016) 374:1123–33. 10.1056/NEJMoa1510926 26933753 PMC4900689

[26] Mehta N Qamar A Qu L Qasim AN Mehta NN Reilly MP Differential association of plasma angiopoietin-like proteins 3 and 4 with lipid and metabolic traits. *Arterioscler Thromb Vasc Biol.* (2014) 34:1057–63. 10.1161/ATVBAHA.113.302802 24626437 PMC4104779

[27] Dijk W Schutte S Aarts EO Janssen IMC Afman L Kersten S. Regulation of angiopoietin-like 4 and lipoprotein lipase in human adipose tissue. *J Clin Lipidol.* (2018) 12:773–83. 10.1016/j.jacl.2018.02.006 29555209

[28] Aryal B Price NL Suarez Y Fernandez-Hernando C. ANGPTL4 in metabolic and cardiovascular disease. *Trends Mol Med.* (2019) 25:723–34. 10.1016/j.molmed.2019.05.010 31235370 PMC6779329

[29] Cheung KJ Tzameli I Pissios P Rovira I Gavrilova O Ohtsubo T Xanthine oxidoreductase is a regulator of adipogenesis and PPARgamma activity. *Cell Metab.* (2007) 5:115–28. 10.1016/j.cmet.2007.01.005 17276354

[30] Sautin YY Nakagawa T Zharikov S Johnson RJ. Adverse effects of the classic antioxidant uric acid in adipocytes: NADPH oxidase-mediated oxidative/nitrosative stress. *Am J Physiol Cell Physiol.* (2007) 293:C584–96. 10.1152/ajpcell.00600.2006 17428837

[31] Hu D Yang Y Peng DQ. Increased sortilin and its independent effect on circulating proprotein convertase subtilisin/kexin type 9 (PCSK9) in statin-naive patients with coronary artery disease. *Int J Cardiol.* (2017) 227:61–5. 10.1016/j.ijcard.2016.11.064 27846466

[32] Rule AD Larson TS Bergstralh EJ Slezak JM Jacobsen SJ Cosio FG. Using serum creatinine to estimate glomerular filtration rate: accuracy in good health and in chronic kidney disease. *Ann Intern Med.* (2004) 141:929–37. 10.7326/0003-4819-141-12-200412210-00009 15611490

[33] Nakagawa T Tuttle KR Short RA Johnson RJ. Hypothesis: fructose-induced hyperuricemia as a causal mechanism for the epidemic of the metabolic syndrome. *Nat Clini Pract Nephrol.* (2005) 1:80–6. 10.1038/ncpneph0019 16932373

[34] Tjeerdema N Georgiadi A Jonker JT van Glabbeek M Alizadeh Dehnavi R Tamsma JT Inflammation increases plasma angiopoietin-like protein 4 in patients with the metabolic syndrome and type 2 diabetes. *BMJ Open Diabetes Res Care.* (2014) 2:e000034. 10.1136/bmjdrc-2014-000034 25512873 PMC4265148

[35] Bougarne N Weyers B Desmet SJ Deckers J Ray DW Staels B Molecular actions of PPARα in lipid metabolism and inflammation. *Endocr Rev.* (2018) 39:760–802. 10.1210/er.2018-00064 30020428

[36] Huang RL Teo Z Chong HC Zhu P Tan MJ Tan CK ANGPTL4 modulates vascular junction integrity by integrin signaling and disruption of intercellular VE-cadherin and claudin-5 clusters. *Blood.* (2011) 118:3990–4002. 10.1182/blood-2011-01-328716 21841165

[37] Smart-Halajko MC Kelley-Hedgepeth A Montefusco MC Cooper JA Kopin A McCaffrey JM ANGPTL4 variants E40K and T266M are associated with lower fasting triglyceride levels in Non-Hispanic White Americans from the Look AHEAD Clinical Trial. *BMC Med Genet.* (2011) 12:89. 10.1186/1471-2350-12-89 21714923 PMC3146919

[38] Dijk W Kersten S. Regulation of lipoprotein lipase by Angptl4. *Trends Endocrinol Metab.* (2014) 25:146–55. 10.1016/j.tem.2013.12.005 24397894

[39] Olshan DS Rader DJ. Angiopoietin-like protein 4: a therapeutic target for triglycerides and coronary disease? *J Clin Lipidol.* (2018) 12:583–7. 10.1016/j.jacl.2018.01.012 29548670

[40] Lichtenstein L Berbee JF van Dijk SJ van Dijk KW Bensadoun A Kema IP Angptl4 upregulates cholesterol synthesis in liver via inhibition of LPL- and HL-dependent hepatic cholesterol uptake. *Arterioscler Thromb Vasc Biol.* (2007) 27:2420–7. 10.1161/ATVBAHA.107.151894 17761937

[41] Vekic J Jelic-Ivanovic Z Spasojevic-Kalimanovska V Memon L Zeljkovic A Bogavac-Stanojevic N High serum uric acid and low-grade inflammation are associated with smaller LDL and HDL particles. *Atherosclerosis.* (2009) 203:236–42. 10.1016/j.atherosclerosis.2008.05.047 18603253

[42] Smart-Halajko MC Robciuc MR Cooper JA Jauhiainen M Kumari M Kivimaki M The relationship between plasma angiopoietin-like protein 4 levels, angiopoietin-like protein 4 genotype, and coronary heart disease risk. *Arterioscler Thromb Vasc Biol.* (2010) 30:2277–82. 10.1161/ATVBAHA.110.212209 20829508 PMC3319296

[43] Nilsson SK Anderson F Ericsson M Larsson M Makoveichuk E Lookene A Triacylglycerol-rich lipoproteins protect lipoprotein lipase from inactivation by ANGPTL3 and ANGPTL4. *Biochim Biophys Acta.* (2012) 1821:1370–8. 10.1016/j.bbalip.2012.06.003 22732211

[44] Graham MJ Lee RG Brandt TA Tai L-J Fu W Peralta R Cardiovascular and metabolic effects of ANGPTL3 antisense oligonucleotides. *N Engl J Med.* (2017) 377:222–32. 10.1056/NEJMoa1701329 28538111

